# Two‐Phase Material Shape Optimization of an Additively Manufactured Integrated Metal and Ceramic Resin Implant‐Supported Dental Crown

**DOI:** 10.1002/cnm.70095

**Published:** 2025-09-10

**Authors:** Joseph Way, Sanjay Joshi

**Affiliations:** ^1^ Department of Additive Manufacturing and Design Pennsylvania State University University Park Pennsylvania USA; ^2^ Department of Industrial and Manufacturing Engineering Pennsylvania State University University Park Pennsylvania USA

**Keywords:** 3D printing, dental implant restoration, shape‐optimization

## Abstract

The screw‐retained implant‐supported crown is a durable, aesthetic restoration, but debonding between the crown and abutment remains a challenge to survivability. The purpose of this work was to devise an abutment shape that can be embedded into the crown while the crown is being additively manufactured. The result was a mechanically retained, no‐adhesive abutment and crown unit that is mounted to the implant fixture. To generate the best internal shape for the new restoration design concept, a shape optimization method was developed using nTop software with the objective of pursuing low structural compliance (maximizing stiffness), withstanding mastication loads, and complying with the unique manufacturing constraints of the proposed design. The optimization results showed a 39% and 51% reduction in structural compliance for molar and incisor restorations. Validation finite element analysis (FEA) on the molar restoration was accomplished for comparison of the initial, optimized, and traditional Ti‐Base screw‐retained designs. Under vertical and angled loads, the optimized design reduced maximum Von Mises stress by 38% compared with the traditional design, and under upwards load, the optimized design reduced maximum principal shear strain along the abutment‐crown joint boundary by 67%. A successful prototype was created using a stereolithography (SLA) printer for fit and form testing. The design concept in this study showed promise as an alternate method to join the two components, while removing the debonding failure mode and maintaining aesthetics and strength. This may offer a more suitable screw‐retained restoration option for patients with constraints such as small interocclusal space.

## Graphical abstract

This work focuses on developing an abutment shape that can be embedded into the crown while the crown is being 3D‐printed, resulting in a mechanically retained, no‐adhesive unit that is mounted to the implant fixture. A shape optimization method was developed to derive the best internal geometry for this design, with the algorithm maximizing stiffness while complying with the unique manufacturing constraints of the concept. Results show improved properties from optimization, lower stresses, and successful prototyping using 3D‐printing.

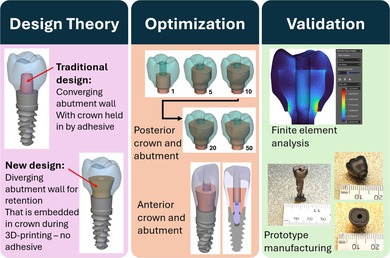

## 1 Introduction

**FIGURE 1:**
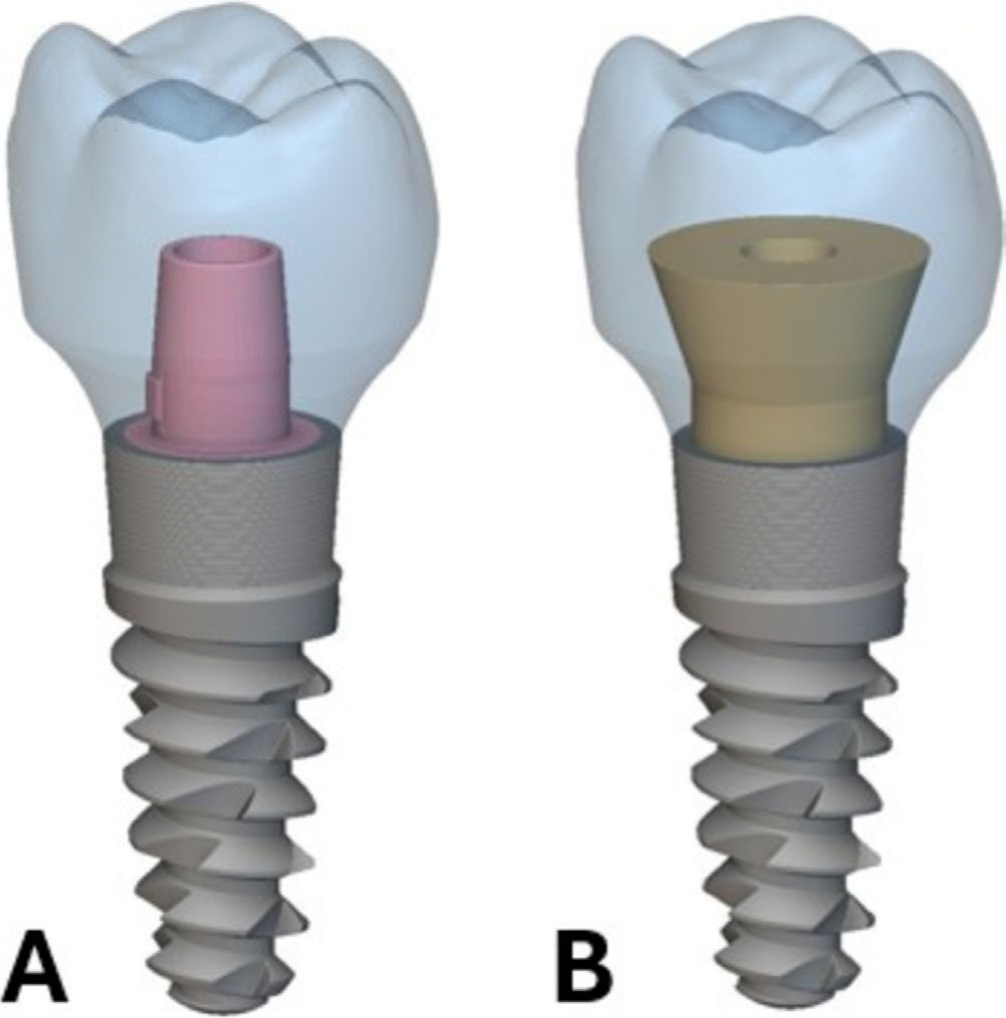
Single implant supported, screw‐retained restoration designs. (A) Conventional design using an extra‐oral applied adhesive to join the crown (blue) and abutment (pink). (B) New design mechanically embedding the abutment (gold) inside the crown (blue).

Dental implants and crowns are widely applied dental prostheses used to replace the form, fit, and function of edentulous groups [1, 2]. The prostheses must resist repeated mastication loads, restore aesthetic form, and correctly fit with the surrounding morphology [3]. Dental implant restorations usually follow two main categorizations: screw‐retained restorations and cement‐retained restorations. Most treatments with either type often have similar outcomes. However, screw‐retained restorations offer superior retrievability and ease of repair, while also being indicated for cases with small interocclusal space due to their shorter minimum height. The risk of biological complications from excess intra‐oral cement with cement‐retained restorations should also be recognized [4]. Many opt for the screw‐retained restoration based on these considerations. Nonetheless, the various clinically documented designs or types of screw‐retained restorations possess their own associated challenges. In the case of Ti‐Base stock abutment or custom abutment style screw‐retained restorations that are bonded extra‐orally to a ceramic or resin crown, a critical failure mode exists at this interface between the abutment and crown since it is held by a weaker bonding or luting material—consequently posing a risk for debonding [5, 6, 7, 8]. Another type of design consisting of a single‐piece abutment and crown that is milled or cast from metal, while lacking a weak interface between materials, presents major aesthetic drawbacks for the patient [9]. The purpose of this research is to develop an alternate screw‐retained restoration design that eliminates the debonding failure mode by mechanically embedding the abutment inside of the crown while still offering the aesthetic benefits of a ceramic‐filled resin crown on the exterior. Diverging surfaces are used to hold the abutment inside and prevent separation, and additive manufacturing (AM) is proposed as an effective way to join the two components during the printing process. Figure 1A,B shows examples of converging and diverging screw‐retained abutments (hole through crown not pictured, for clarity). Figure 1A depicts a Ti‐Base style converging abutment that is inserted into the intaglio surface of the crown and secured with adhesive extra‐orally. Figure 1B shows the new diverging design, where the abutment is embedded in the crown without the use of adhesive during the 3D printing of the crown. The null hypothesis of this study is that the new design does not offer any mitigation of the debonding failure mode or survivability benefits.

Metals are proven to have superior strength, ductility, and fatigue resistance for the demanding conditions of dental prostheses, but they alone do not meet the aesthetic standards that patients demand to be used as a standalone single‐piece crown and abutment for screw‐retained restorations [9]. Ceramic materials demonstrate aesthetic properties and sufficient flexural strengths and moduli for implant restoration applications, specifically as a single‐piece crown and abutment. However, these materials are highly brittle and can often experience fatigue failure under the repetitive, concentrated tensile loads found in the abutment component, shortening the lifespan of the prosthesis [10]. While ceramic‐filled resin crowns and abutments made with AM exhibit good fatigue resistance and aesthetic appearance, they are often too compliant to resist and survive peak mastication loads in the posterior region if built as a single‐piece abutment and crown screw‐retained restoration [11]. The inherent benefits and drawbacks of these three restorative material classes make the implant‐supported restoration unsuitable to be entirely made from only one material alone. Therefore, ceramics and ceramic‐filled resins are often used in conjunction with metals to construct the screw‐retained restoration; the metal is placed in critical load regions, such as the abutment, to enhance the survivability of the prothesis [12]. However, adding a second material dictates the need for a method of joining them together, which is traditionally accomplished using an adhesive or bonding material in both cement‐retained (intra‐orally) and screw‐retained (extra‐orally) [13]. However, the interface between the metal abutment and the crown in either type, secured by adhesive, can debond under loading conditions that the patient places on it [5]. Furthermore, a number of factors have been found to influence the strength of the adhesive bond, such as the cleaning procedure and preparation of the bonding surfaces [14, 15]. A review of literature pertaining to single‐implant restorations with metal‐ceramic bonds sheds light on the commonality of this failure mode. A study on the 3‐year survivability of this type of prosthesis found a 5.7% failure incidence rate of just debonding between materials alone [16]. Another 6‐year survivability study reported a 1.3% incidence of debonding between the ceramic and Ti‐Base abutments for screw‐retained restorations [17]. The same debonding issue can also be common in implant‐supported fixed partial denture restorations, with one study reporting that 58% of failures in a 12‐year retrospective study and 46% of failures in a 5‐year retrospective study occurred by debonding of the ceramic resin from the metal framework [18, 19]. It is clear that in screw‐retained restorations and many other types of dental prostheses, mastication loads cause the bond between two materials to fail at relevant rates.

Therefore, the logical solution to the debonding issue is to mechanically embed the metal abutment in the crown instead of using adhesive. AM offers an opportunity to embed structures during the printing process and requires little manual input to accomplish [20]. One study took this approach by making a latticed metal framework with laser power bed fusion (LPBF) and then used an overprinting technique, where resin was cured additively around the metal resin shell with laser stereolithography (SLA) for the dental prosthesis. Prototype manufacturing was attempted using the lattice as mechanical retention for the ceramic resin shell, but results were not reported [21]. While overprinting likely offers the most design freedom for the embedded metal abutment, most of the AM processes today struggle with overprinting. A few prototype printers are being explored currently but are not proven for overprinting on a complex structure [22].

Given the challenges with this technique, another method for embedding the metal abutment during an AM process is proposed in this paper. The metal abutment can be designed without any undercuts so that it can be inserted into the ceramic resin crown during SLA printing. The ceramic resin printing process can be paused at the opportune layer, the metal can be inserted, and then the printing process can be resumed where it prints over the top of the metal abutment. This yields a mechanically retained abutment with no adhesive needed. However, a few design constraints must be considered. First, the metal abutment must be designed with a diverging shape starting from its interface with the implant. The diverging shape, as opposed to straight or converging, does not allow the metal abutment to fall out of the bottom of the ceramic resin shell when it is inserted. There also cannot be any undercuts on the surface of the diverging shape, which would make it impossible to fully insert the abutment. Furthermore, the abutment must have a top surface that is parallel to the build plane so that printing can be resumed over the metal abutment without collisions with the printing machine. Finally, the ceramic resin crown around the metal abutment must retain a minimum thickness to mitigate chipping [23].

These design constraints drive uncertainty in the ideal shape of the metal abutment since most conventional metal abutment designs are not similarly restricted. The design space should then be explored computationally to achieve a suitable design. Finite element analysis (FEA) has been shown to provide suitable performance assessments for various dental prostheses, including implant‐supported crowns, that are subject to mastication loads [24]. Design optimization strategies have found application throughout many engineering problems, but little research has focused specifically on dental or even implant crown applications [25]. Park et al. applied topology optimization studies to complete denture frameworks to increase structural efficiency [26]. Chen et al. utilized a bi‐directional evolutionary structural efficiency (BESO) algorithm for the denture base of a removable partial denture to optimize the distribution of contact pressure on oral mucosa [27]. Zhang et al. also applied the BESO algorithm to a dental crown‐pontic joint to improve the fracture resistance [28]. Fractional factorial parametric optimization studies were performed on dental implants to determine ideal geometric parameters [29, 30]. A two‐step optimization was used to reduce the peak stresses in a cantilever bridge and place structural fibers in the prosthesis [31]. A well‐developed computational optimization strategy can be adapted to the patient and quickly converge on a high‐performing implant crown design that will prevent debonding complications.

## 2 Methods

**FIGURE 2:**
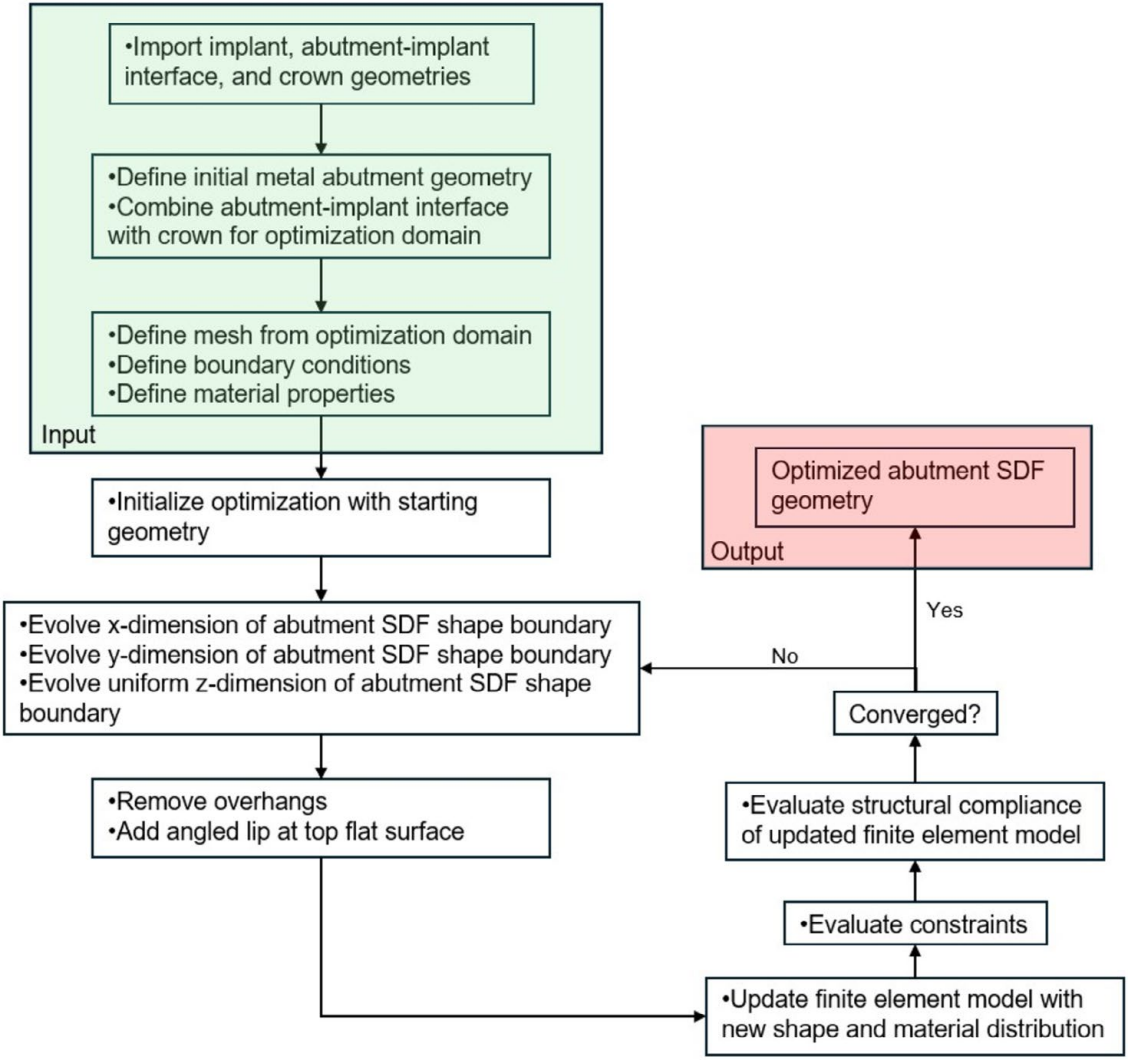
Summary of optimization pathway used in the study.

To develop a high‐performance design that mitigates the debonding failure mode while utilizing the additive manufacturing methods described, a gradient‐based parametric optimization study that simultaneously obeys multiple manufacturing constraints was created. FEA is used to validate the final design and compare it to a conventional screw‐retained design for an implant‐supported dental crown. The process was then wrapped into a computational design workflow for a rapid, repeatable process that only requires boundary conditions and an initial guess for parameters as inputs. Sample outcomes from the computational design workflow were additively manufactured and tested for fit and form. Figure 2 graphically depicts a summary of the optimization process developed in this study.

A variety of shape optimization methods are commonly used today, such as the solid isotropic material with penalization (SIMP) method or the level‐set method [32]. A slight variation of the level‐set method was set up using a field‐based modeling software (nTop) with an embedded gradient‐based algorithm. Within the tool, three scalar fields in the Cartesian axes directions were established as optimizable parameters that modify the abutment shape. These three scalar fields influence changes to the localized scaling and warping of the three Cartesian axes and subsequently modify the initial signed distance field (SDF) representation of the shape. The shape is then iteratively remapped based on the three corresponding parameters to create the updated SDF shape.

As previously mentioned, there is a manufacturing constraint on SLA 3D printers dictating that the top surface of the abutment is flat [33]. Therefore, the field parameter with a normal vector to the top surface is constrained so that it will only evolve the boundary uniformly. An example of this flat top is shown in Figure 1B. In order to constrain the parameter in this way without degrading the optimization process, the scalar value of the unconstrained parameter field is sampled with random points within the initial SDF shape. Then, the scalar values of the unconstrained parameter field are remapped to the specific scalar value at the perpendicular axis locations of the points. The field is left to vary along the plane created from the normal direction to the top surface. A list of constrained scalar fields is produced from this operation. Finally, the list of fields is averaged to produce a final field that meets the constraint and prevents the gradient optimization from settling in a local minimum. A second manufacturing constraint must also be incorporated into the optimization process to prevent the possibility of undercuts forming on the sides of the abutment. Some research has been completed on removing overhangs during optimization, mainly for the application of demolding injection molded parts [34, 35]. Building on this, a voxel grid and a ray‐casting subroutine are used to identify the undercuts. Then, these regions are removed from the abutment. An example of a diverging abutment shape with no undercuts is also shown in Figure 1B. A final manufacturing constraint dictating that the crown retains a minimum 1 mm thickness is added to the optimization to ensure that a sufficient thickness of ceramic resin material remains around the metal abutment.

**FIGURE 3:**
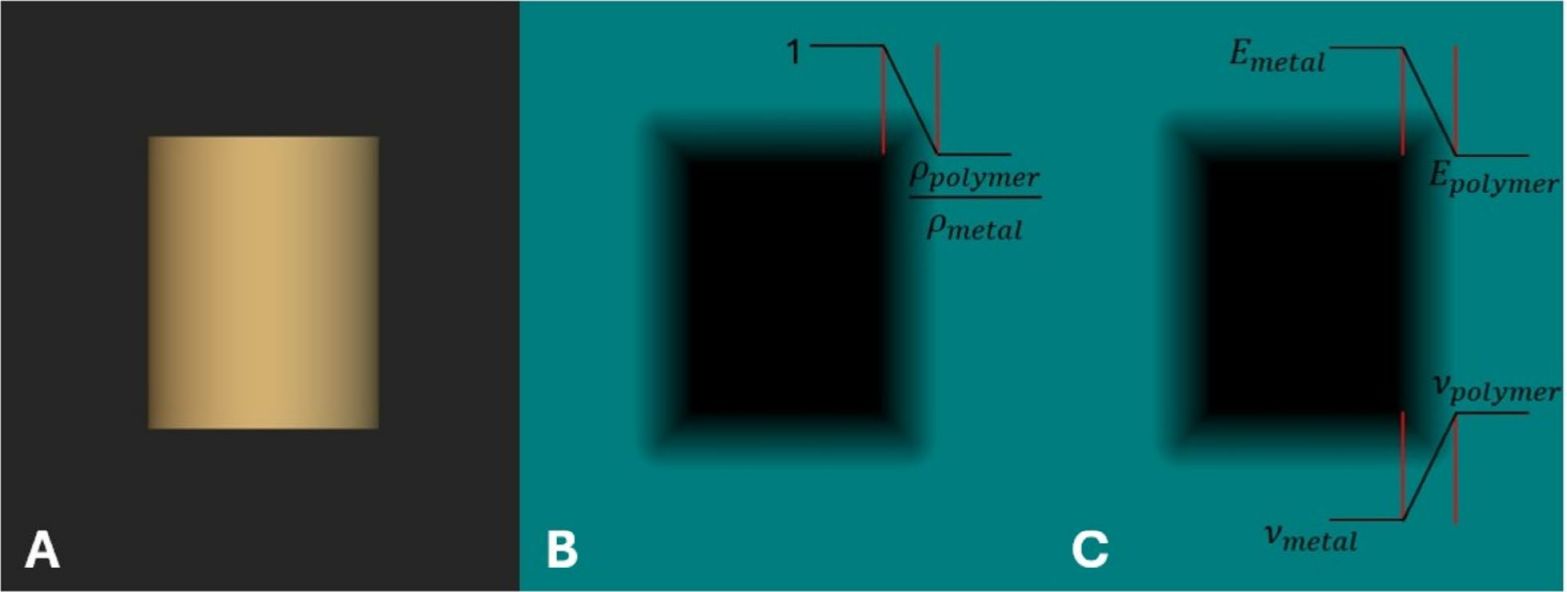
Optimization models definitions. (A) Geometry model. (B) Porosity model with relative density. (C) Material model with elastic modulus and Poisson's ratio.

After the shape evolution and constraints were applied in each step of the evolution, geometry, porosity, and material properties models were added in the optimization software. The geometry model is set to be the SDF representation of the shape evolution of only the metal abutment, as seen in the example geometry of Figure 3A. Additionally, a small, angled lip is added to the edge of the top surface at each iteration, so that the abutment always retains some mechanical retention within the crown. After the optimization is finished, simple Boolean operations can be used to extract the corresponding crown shape. The porosity model is set as a scalar field, where the SDF representation of the current‐step metal abutment takes a relative density of one and the remaining volume within the design domain takes on the relative density of the crown material to the metal abutment material. Figure 3B shows the porosity representation, and the linear ramp function transition from the abutment to the crown region. The material model takes a similar approach to the porosity model but instead assigns the elastic modulus and Poisson's ratio of each material to the corresponding locations. To maintain mesh independence from the porosity and material distributions, a transition region linear ramp function between abutment and crown material properties is applied and set to 1.5 times the average edge length of the mesh. Figure 3B,C show a graph of the linear transition regions between the crown and abutment material and density properties. The dark region represents uniform abutment properties, the blue region represents uniform crown properties, and the faded region represents the transition. These three models, joined with the constraints and shape evolution process, describe the overall data necessary for the optimization process used in this study.

**FIGURE 4:**
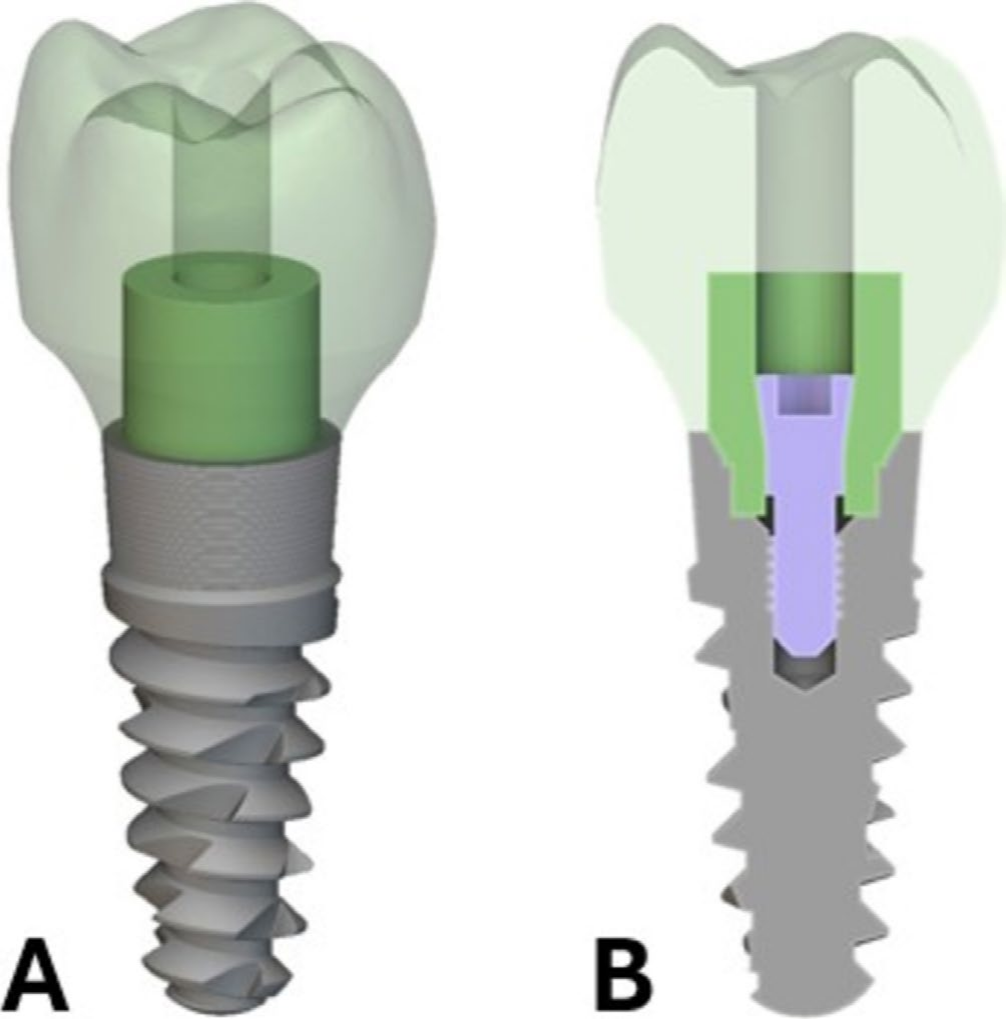
Crown, initial abutment shape, implant, fastener, and fastener hole geometries inputs used in optimization pathway. (A) 3D projection view. (B) Cross‐section view.

The initial geometry used for the implant was a tapered internal implant taken from GrabCAD 3D model repository [36]. Then a model for the crown region of a mandibular right first molar and a maxillary central incisor was obtained from the Invisalign ClinCheck software. Implicit modeling was used to loft the underside of the molar and incisor crowns to the top of the implant platform for a smooth emergence profile and create the region between the implant and the clinical crown. The occluso‐cervical length for the molar crown and incisor crown was measured as 7.68 mm and 13.22 mm. The bucco‐lingual length for the molar crown and facial‐lingual length for the incisor crown was measured as 9.78 mm and 7.56 mm. The mesio‐distal length for the molar crown and incisor crown was measured as 10.68 mm and 9.21 mm. Finally, a screw channel measuring 2.4 mm in diameter used for inserting the screw that joins the abutment and implant was cut through the entire region (crown and abutment) of the molar and incisor to make screw‐retained restorations. The combination of the abutment‐implant interface, emergence profile, and tooth geometry was joined to create the design domain for the optimization. The abutment was assigned as Ti‐6Al‐4 V material, and both crowns were assigned as NextDent C&B MFH 3D printed ceramic‐filled resin. The implant and fastener geometries were used for visualization, but not for any part of the optimization. Figure 4A,B show a 3D projection and cross‐section views of these key regions of the design space and the implant fixture that were used for the optimization. The molar tooth geometry (as an example) is shown in light green, and the abutment fastener in purple. The starting geometry for the abutment in dark green was chosen as a simple cylinder for both the molar and incisor optimization, but any shape that fits within the design domain could be input to the optimization algorithm as a starting guess.

**FIGURE 5:**
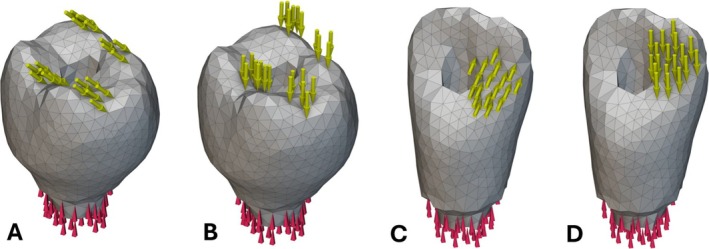
Boundary conditions. (A) 200 N 45‐degree angled load on molar. (B) 200 N vertical load on molar. (C) 200 N 45‐degree angled load on incisor. (D) 200 N vertical load on incisor.

The design domain geometry was then meshed for the finite element model using a tetrahedral volume mesh. A mesh edge length of 0.75 mm was chosen to balance fidelity with efficiency, which resulted in a mesh containing 2069 nodes and 10,179 elements. The nodes at the bottom of the abutment were constrained to have no displacement, representing the abutment connected to the fixture. A combined loading condition was used during the optimization, comprised of a vertical load and an angled load applied at the same time. The vertical load was oriented parallel to the long axis of the implant with a magnitude of 200 N. The angled load was set to 45 degrees from the vertical load direction and pointed buccally with a magnitude of 200 N [37, 38]. On the molar crown, the vertical and angled loads are distributed across the distal marginal ridge, distobuccal cusp, mesiobuccal cusp, and central fossa. On the central incisor crown, the vertical and angled loads are distributed onto the mesial marginal ridge. Both loads and the mesh for the molar optimization are shown in Figure 5A,B, along with the displacement restraints at the bottom of the mesh. Both loads and the mesh for the incisor optimization are shown in Figure 5C,D, also with the displacement restraints at the bottom of the mesh.

For the optimization, the specific material properties shown in Table 1 are assigned to the metal and ceramic resin portions of the geometry. The optimization was initialized and set to a maximum iteration number of 50. The move limit was set to 20% of the maximum shape parameter change of 10 mm. The objective of the optimization was set to minimize structural compliance. This objective was chosen to drive towards a structure that would not bend or flex under mastication loads. A maximum stress constraint of 590 MPa was added to prevent stress from exceeding a 1.5 factor of safety limit on the yield strength of the Ti‐6Al‐4 V abutment [40].

**TABLE 1:** Material properties used in optimization and finite element studies on initial, optimized and traditional screw‐retained models.

Component	Material	Density (kg/m^3^)	Elastic Modulus (Gpa)	Poisson's ratio	References
Abutment	Ti6Al4V	4540	112.4	0.34	[39, 40, 41]
Ceramic resin crown	NextDent C&B MFH	1184	6.4	0.35	[42, 43]
Adhesive	Resin cement	—	5.0	0.3	[44]

**FIGURE 6:**
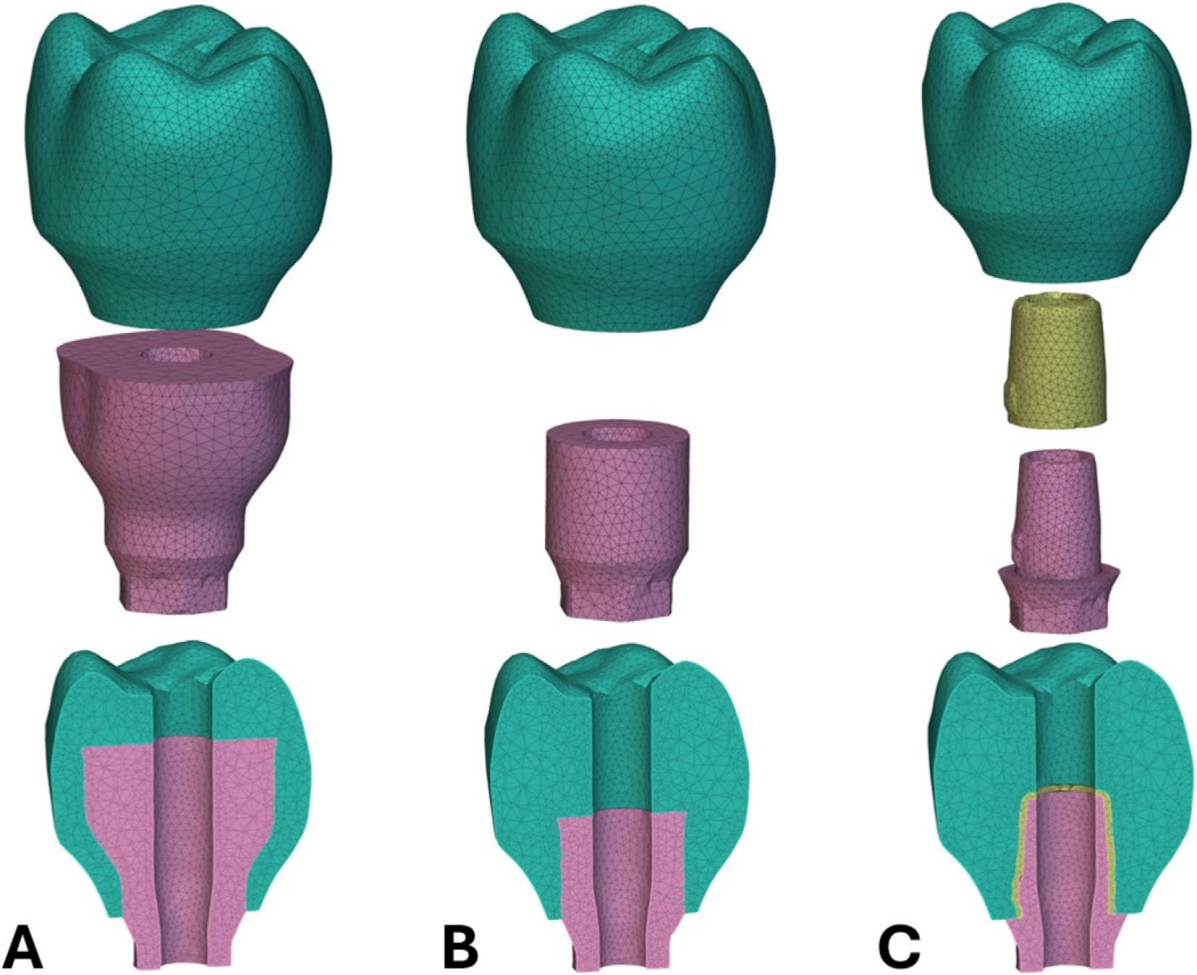
Validation finite element models for screw‐retained restorations. (A) Optimized geometry. (B) Pre‐optimized geometry. (C) Traditional Ti‐Base abutment.

**FIGURE 7:**
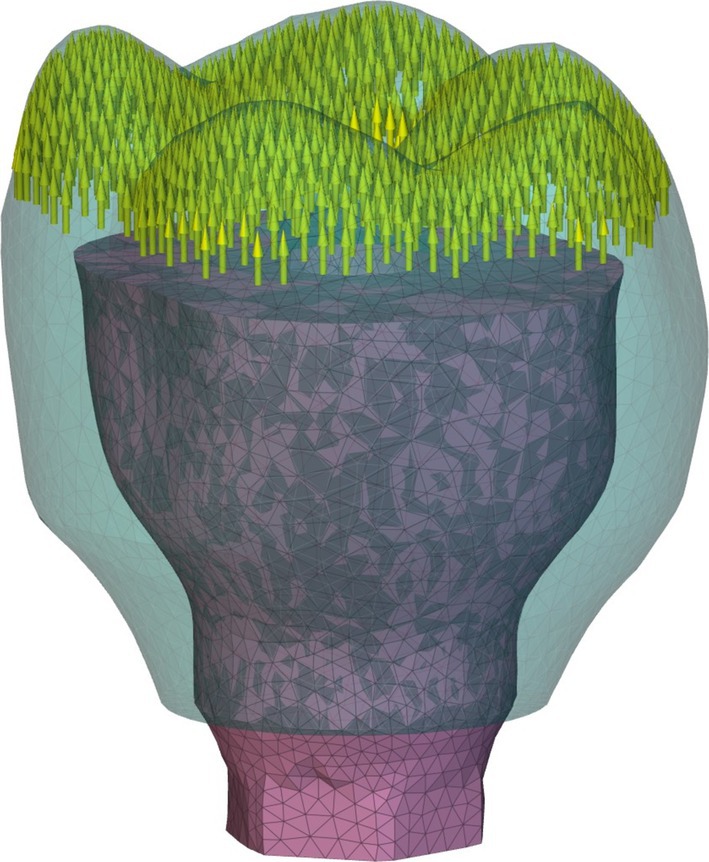
50 N upwards loading condition for validation FEA.

Following the optimization, a series of validation finite element studies were conducted within nTop to assess the performance increase of the optimized design over the initial design and compare it to a conventional screw‐retained Ti‐Base design made of ceramic resin and metal materials. Although using a pure ceramic, such as zirconia, is sometimes a more commonly used molar crown restoration material, a ceramic‐filled resin was used in the validation FEA studies to ensure that a difference in material from the new optimized design was not a confounding variable. Although an optimization routine was completed for the incisor, only the molar geometry was used in the FEA studies. Three studies were set up, consisting of the optimized design, the initial design before optimization, and the traditional screw‐retained Ti‐Base with ceramic resin. The finite element mesh models generated for three studies are shown in Figure 6A–C, showing the components separated and the cross section when they are assembled. The validation analyses for the optimized and initial designs used a two‐component finite element model comprised of the crown and the abutment. A no‐separation contact with a stiffness of 6.08e+04 N/mm was used to represent the metal‐ceramic resin contact between the two components. The validation analysis for the traditional screw‐retained design used a three‐component finite element model comprised of the crown, the abutment, and an adhesive between them [44]. A bonded contact with a stiffness of 5.87e+04 N/mm was used for the metal‐adhesive joint, and a bonded contact with a stiffness of 5.70e+03 N/mm was used for the ceramic‐resin‐adhesive joint [45]. For all three FEA studies, Ti‐6Al‐4 V was chosen as the material for the abutment, and NextDent C&B MFH 3D printed ceramic‐filled resin was chosen for the crown. For the traditional screw‐retained design only, a resin cement was chosen for the extra‐oral adhesive between the crown and abutment. Table 1 shows the material properties used for the three types of materials used in the validation studies. Across the three FEA studies, two sets of loading conditions were analyzed on each design. The first set was the exact same loading conditions used during the optimization, which was the 200 N vertical and 200 N 45‐degree angle load distributed across the distal marginal ridge, distobuccal cusp, mesiobuccal cusp, and central fossa of the molar [37, 38]. The second set of loading conditions was made to simulate upwards pull forces on the crown from a sticky food bolus, directly testing any improvements the optimized design might have over the traditional screw‐retained design for debonding. For this loading condition, a 50 N upwards force was distributed across the occlusal surface of the molar [44]. Figure 7 shows the upwards load applied with the same displacement restraint as before. A static structural analysis was completed on all three models across the two loading conditions for a total of six FEA studies.

**FIGURE 8:**
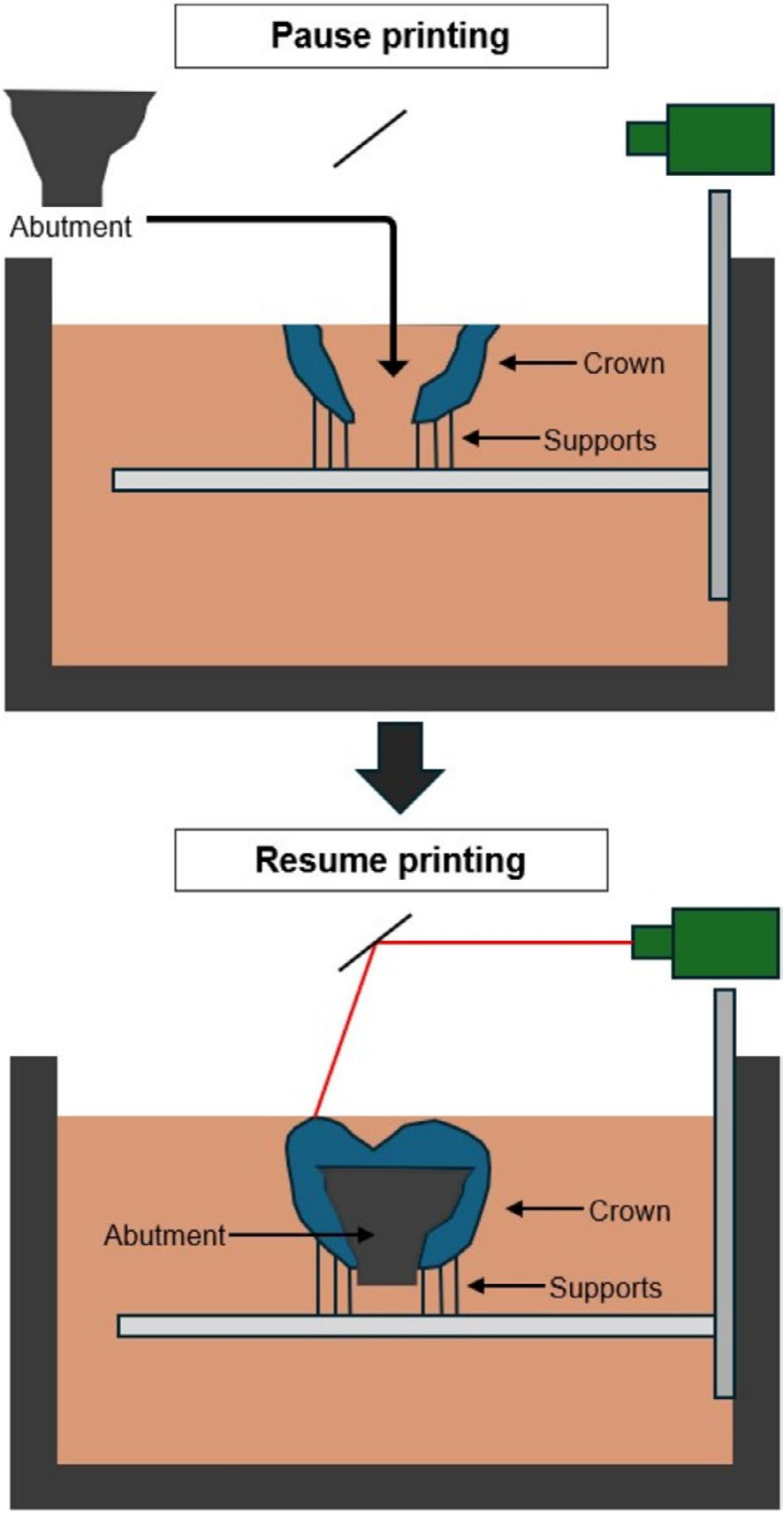
Process for embedding abutment and crown during vat polymerization additive manufacturing.

**FIGURE 9:**
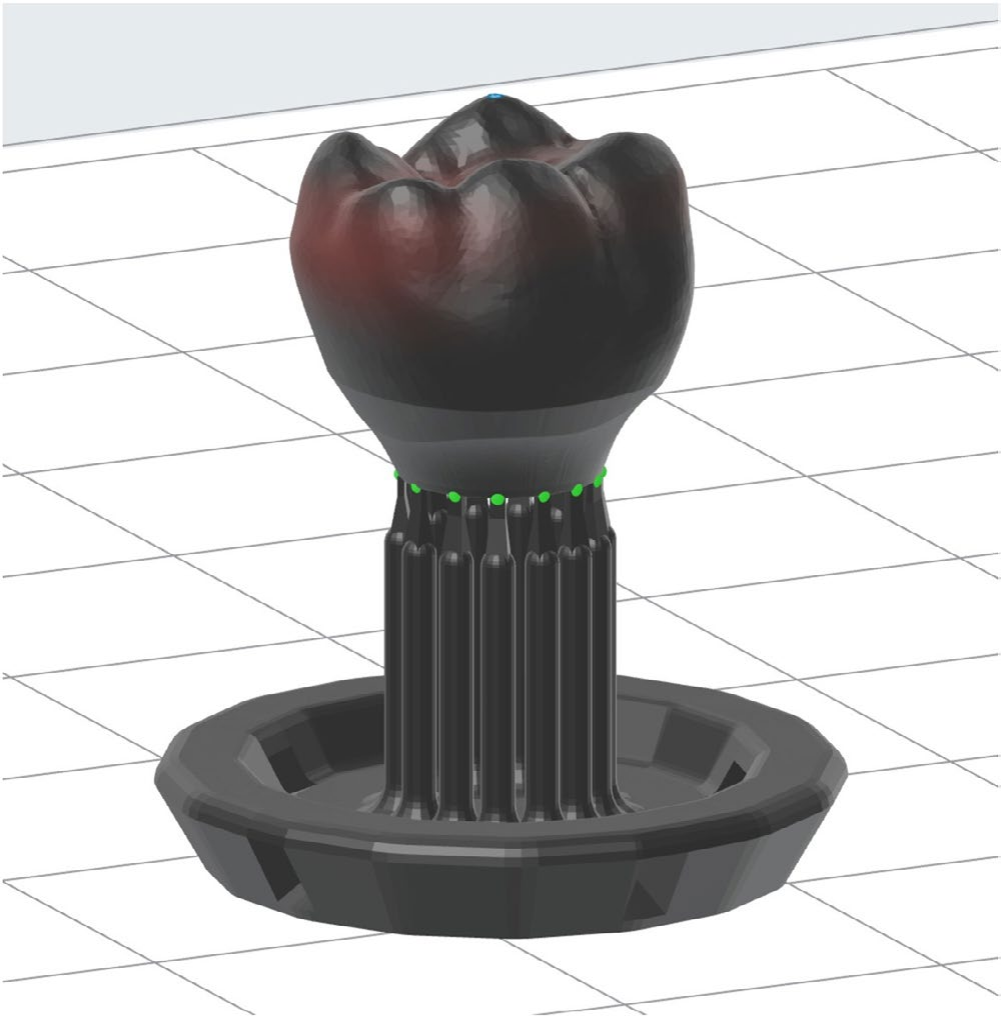
Crown prepared for manufacturing with support structures and raft in 3D printing slicer software.

**FIGURE 10:**
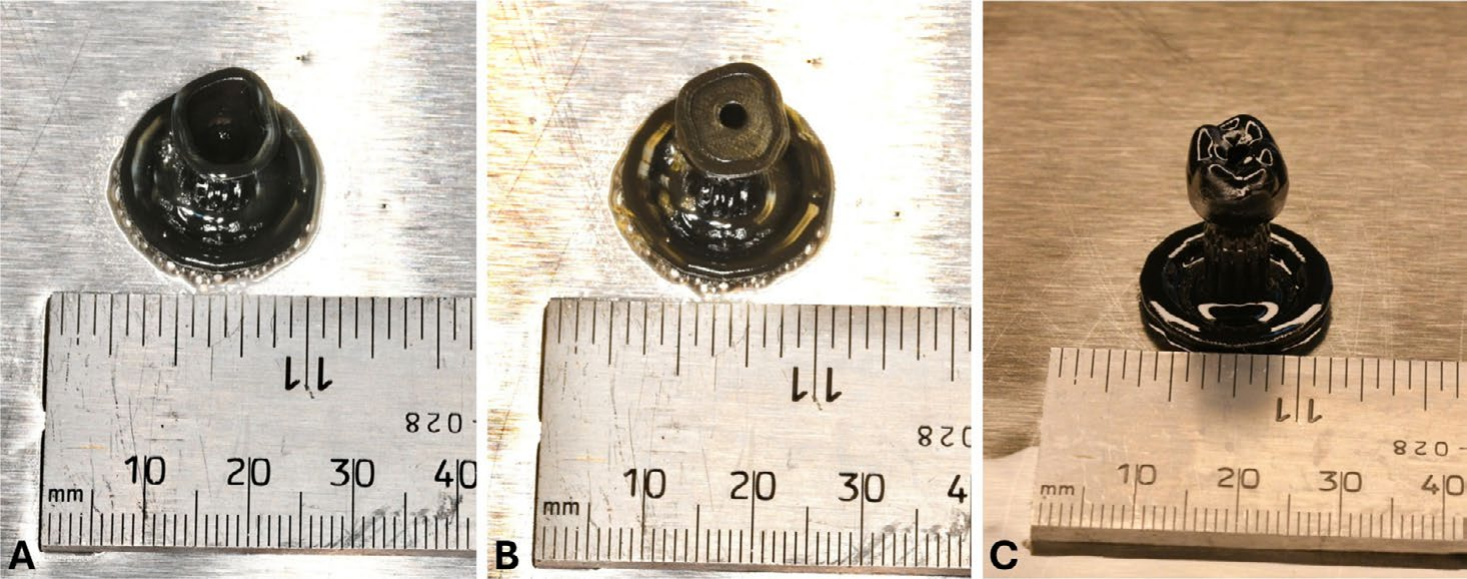
Crown and abutment during 3D printing process. (A) Crown printing paused at the opportune layer. (B) Abutment inserted into the partially completed crown. C, Printing resumed and completed with abutment embedded inside.

Figure 8 shows a diagram of the steps for manufacturing the design. An upright laser SLA machine with ceramic resin was proposed as the most ideal process since the metal abutment could easily be dropped into the ceramic resin shell during the printing process and be held in by gravity. However, an inverted SLA printer was accessible and used in this study without major modification to the process in Figure 8 since gravity did not overcome viscous liquid forces temporarily holding the abutment inverted. A prototype for testing form and fit of the optimized design was printed on a Formlabs Form 3 printer using V4.1 Black Model Resin. First, a model of the abutment was printed normally. A second print was started afterward for the crown, where support structures were added underneath the crown to raise it off the build plate so that the abutment has space to drop in during the printing process. The high viscosity of the resin used in this study and the snug fit between the components were able to hold the abutment in against gravity. Figure 9 shows the crown prepared with support structures in the Preform slicing software. Its height above the build plate was increased to allow the abutment space to drop in. Figure 10A–C show photos taken during 3D printing of the prototype optimized design. Figure 10A depicts the crown print paused before the abutment is inserted at the opportune layer, in this case layer 725 of 863 for the 0.025 mm layer height used during printing. Figure 10B shows the abutment seated in the crown and ready for the printing process to resume. Figure 10C highlights the finished print on the build plate where the upper portion of the crown was printed over the abutment, embedding it inside. After printing was completed, the supports were removed from the part using common hand and power tools such as snippers, a scalpel, and a rotary tool. Sanding and polishing were accomplished to smooth any blemishes from support removal.

## 3 Results

**FIGURE 11:**
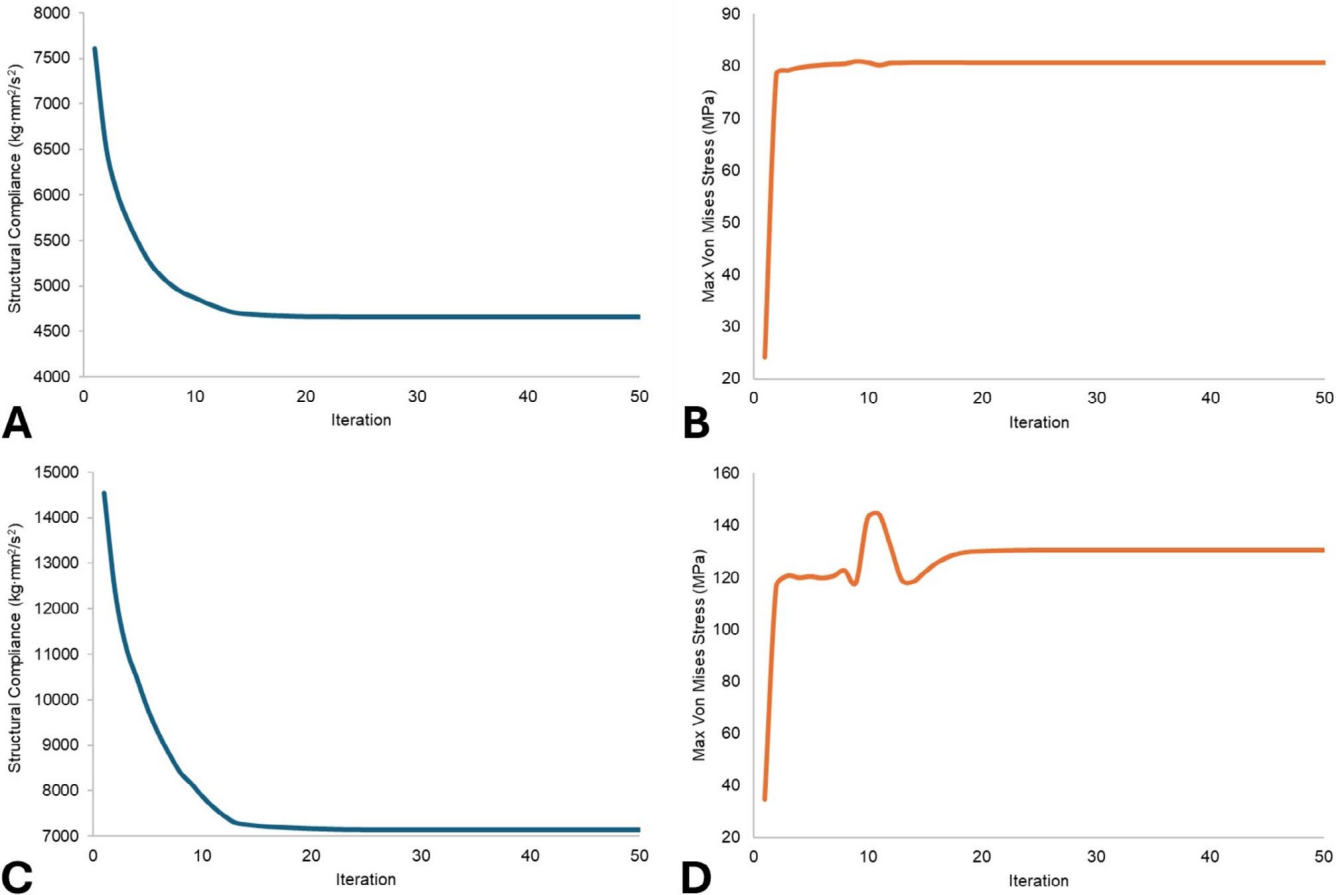
Optimization monitor plots. (A) Structural compliance objective function for molar. (B) Peak Von Mises stress constraint function for molar. (C) Structural compliance objective function for incisor. (D) Peak Von Mises stress constraint function for incisor.

Figure 11A shows a convergence plot of the optimization objective, the structural compliance (inverse of stiffness), for the metal abutment and ceramic resin crown on the molar geometry. The compliance of the design decreased by 39% during the optimization, reduced from 7611.9 to 4655.7 kg·mm^2^/s^2^. Figure 11B shows the maximum Von Mises stress in the design during the optimization of the molar geometry. The upper stress limit was set to 590 MPa; the results show that stress met this constraint and even stayed below the 143 MPa flexural strength of the ceramic resin [40, 42]. Figure 11C then shows the convergence of the objective for the incisor geometry. The compliance of the design in the incisor decreased by 51% during the optimization, falling from 14540.7 to 7140.8 kg·mm^2^/s^2^. Figure 11D again shows the maximum Von Mises stress in the design during the optimization of the incisor geometry. Even though the stress temporarily jumped slightly above 143 MPa around iteration 11, during the remainder of the optimization it stayed below the flexural strength of the ceramic resin [42].

**FIGURE 12:**
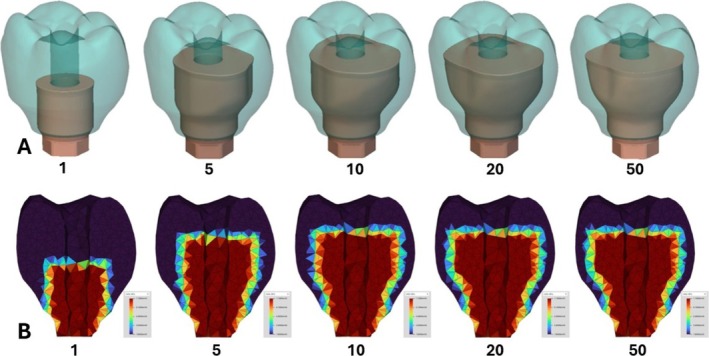
Molar restoration optimization results for steps 1, 5, 10, 20, and 50. (A) Geometric output. (B) Elastic modulus material property distribution output.

**FIGURE 13:**
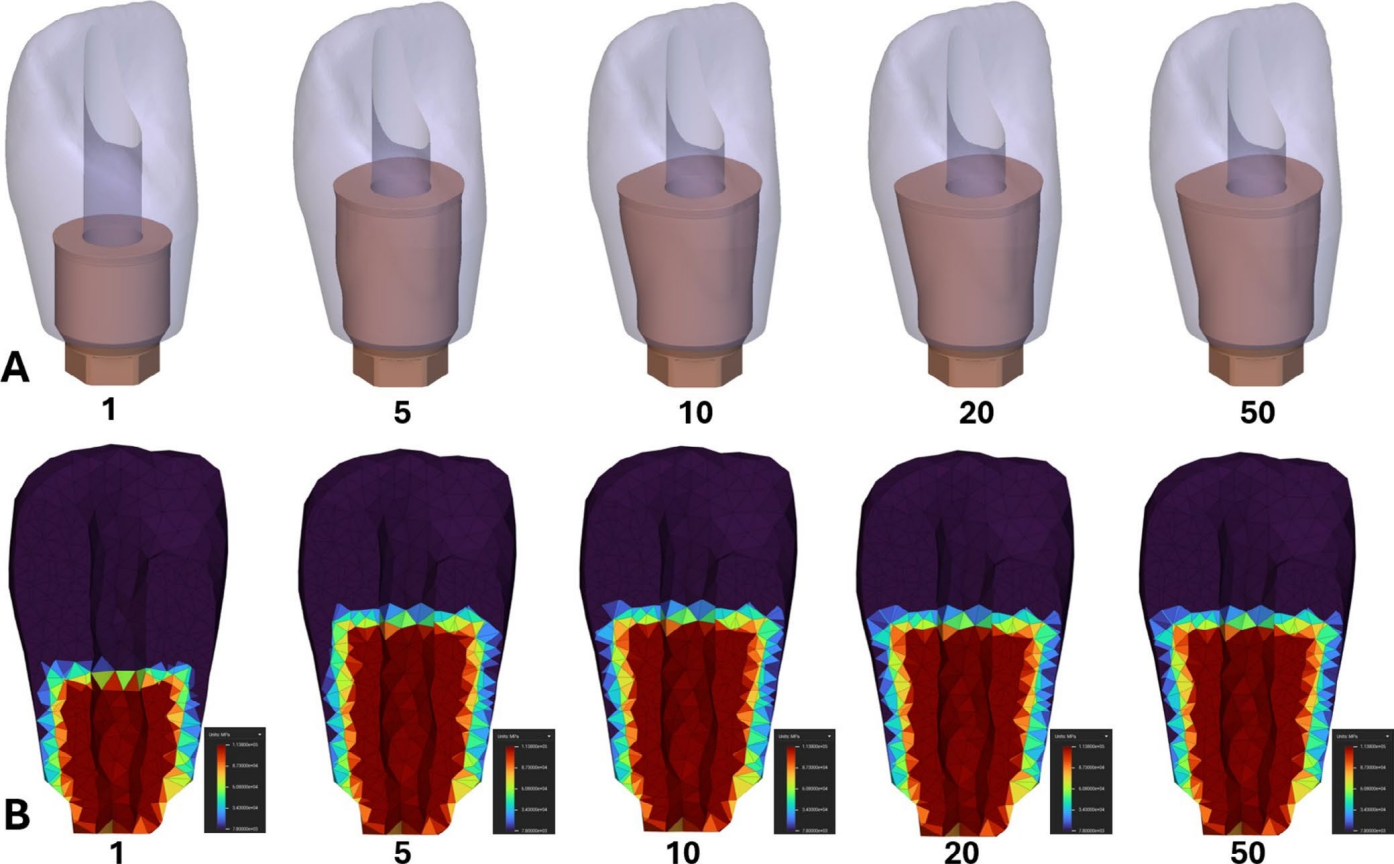
Incisor restoration optimization results for steps 1, 5, 10, 20, and 50. (A) Geometric output. (B) Elastic modulus material property distribution output.

Figure 12A shows the molar geometry evolution and convergence of the metal abutment from the initial starting geometry in Figure 4A towards an optimized shape that fits within the manufacturing constraints. Since overhangs were not allowed and the shape had to maintain a flat top, the shape evolved outward and towards the top of the crown. Figure 12B shows a cross‐section buccal‐lingually of the elastic modulus material distribution through the finite element mesh as the optimization evolved. The transition boundary between the materials can be seen in this figure as the elastic modulus moves from a red color, representing the metal, to the purple color, representing the ceramic resin. Similar results for the incisor geometry are reported in Figure 13A,B.

**FIGURE 14:**
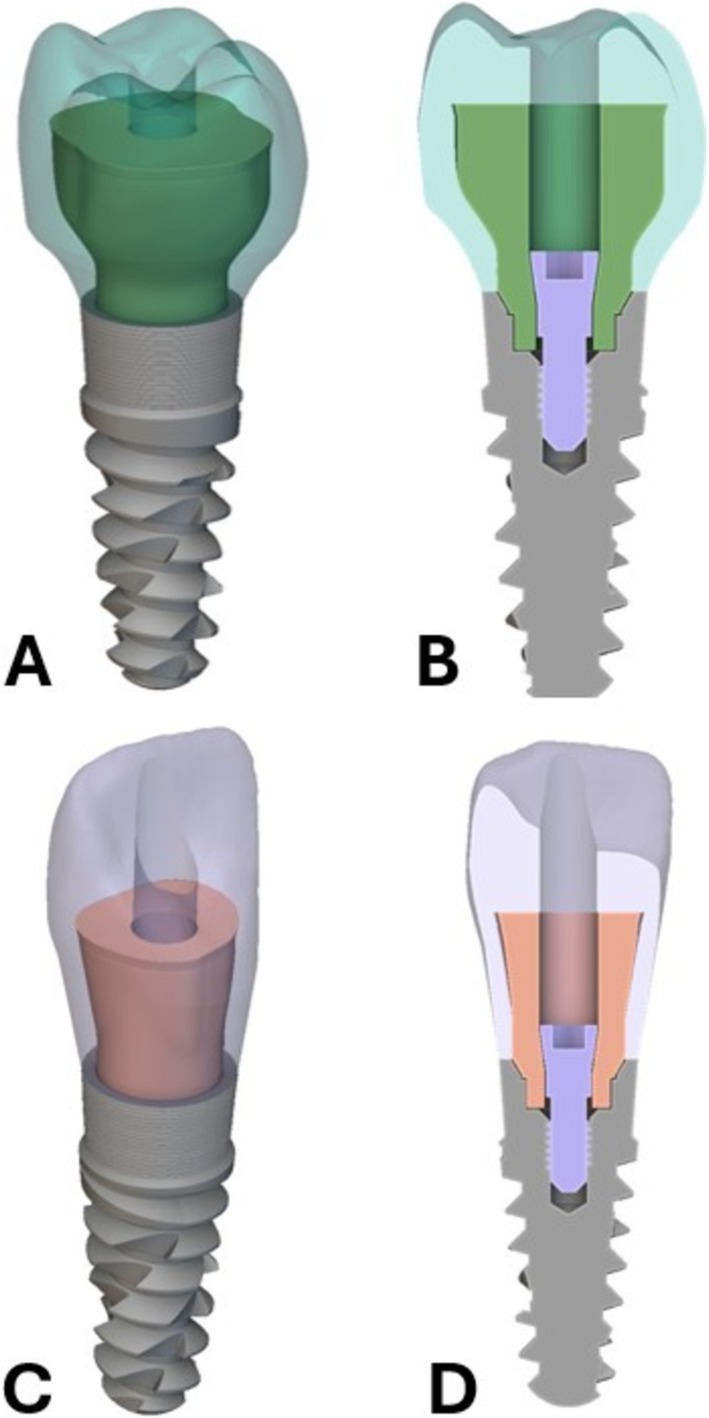
Final optimized screw retained restorations. (A) Mandibular right first molar 3D projection result. (B) Mandibular right first molar cross‐section result. (C) Maxillary right central incisor 3D projection result. (D) Maxillary right central incisor cross‐section result.

Figure 14A,B are then a remake of Figure 4A,B, this time with the optimized abutment and crown shape of the molar. Figure 14C,D show the final optimized shapes for the incisor restoration. The regions of the abutment that interface with the fastener and the implant are preserved during the optimization and are rejoined with the implant geometry. The minimum 1 mm crown thickness constraint can be observed in the section cut from Figure 14B,D. For both tooth restorations (molar and incisor), the optimization loop accepted the unique boundary conditions and developed a complementary abutment shape.

**FIGURE 15:**
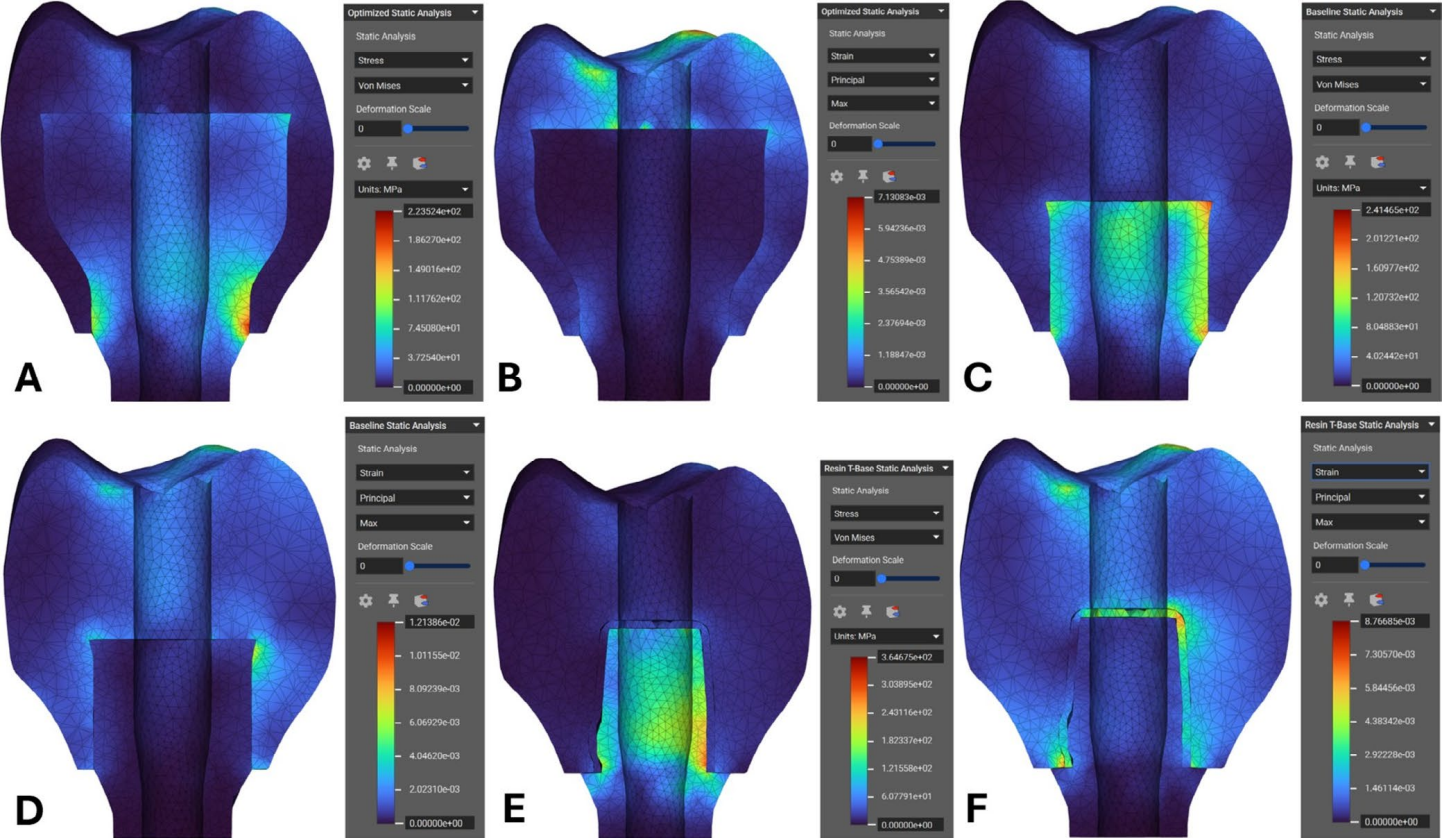
Cross‐section views of static analyses under vertical and angled loads. (A) Von Mises stress analysis of optimized design. (B) Maximum principal strain analysis of optimized design. (C) Von Mises stress analysis of pre‐optimized design. (D) Maximum principal strain analysis of pre‐optimized design. (E) Von Mises stress analysis of traditional Ti‐Base design. (F) Maximum principal strain analysis of traditional Ti‐Base design.

For the FEA validation studies under vertical and angled loads, both the optimized and pre‐optimized designs had similar peak stresses at 223 MPa and 241 MPa (Figure 15A,C). In both models, the peak stresses occur at the base of the crown, where the bending loads push it into the side of the abutment as seen in buccal‐lingual cross sections. The pre‐optimized design also has high stresses at the top corner of the abutment. The maximum principal strain was much lower in the optimized design compared to the pre‐optimized design in Figure 15B,D. The pre‐optimized design has much higher principal strains in the top corner. Both designs show minimal strain across the whole volume of the abutment area, with the optimized design showing slightly less strain between the two. Shifting to the traditional Ti‐Base ceramic resin screw‐retained design, Figure 15E shows a peak stress of 365 MPa, and Figure 15F shows the maximum principal strain. The optimized design had a 38% reduced peak stress and comparable maximum principal strain to the traditional screw‐retained design. Most of the peak stresses in Figure 15E fall around the upright screw channel on the Ti‐Base abutment.

**FIGURE 16:**
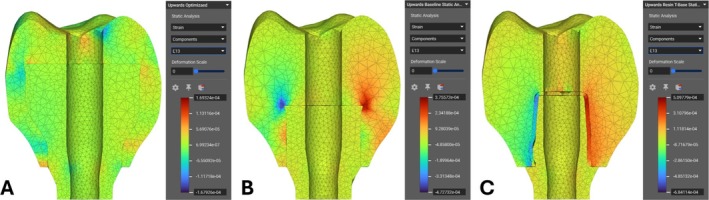
Static analysis cross‐section views of occlusal shear along mesial and distal surfaces under upwards load. (A) Optimized design. (B) Pre‐optimized design. (C) Traditional Ti‐Base design.

Figure 16A–C show the FEA validation study results under upward pull loads for the optimized, pre‐optimized, and traditional Ti‐Base screw‐retained designs. The figures show the shear strain in the occlusal direction in a buccal‐lingual cross‐section view to investigate the effect of the upward pull force on the joint between the restoration components. The traditional Ti‐Base design in Figure 16C had an exceptionally high shear strain in the adhesive that connects the Ti‐Base with the crown compared to the shear strain found anywhere in the optimized design in Figure 16A. There is a 67% decrease in the shear forces found at the boundary between the abutment and the crown from the traditional to the optimized restorations.

**FIGURE 17:**
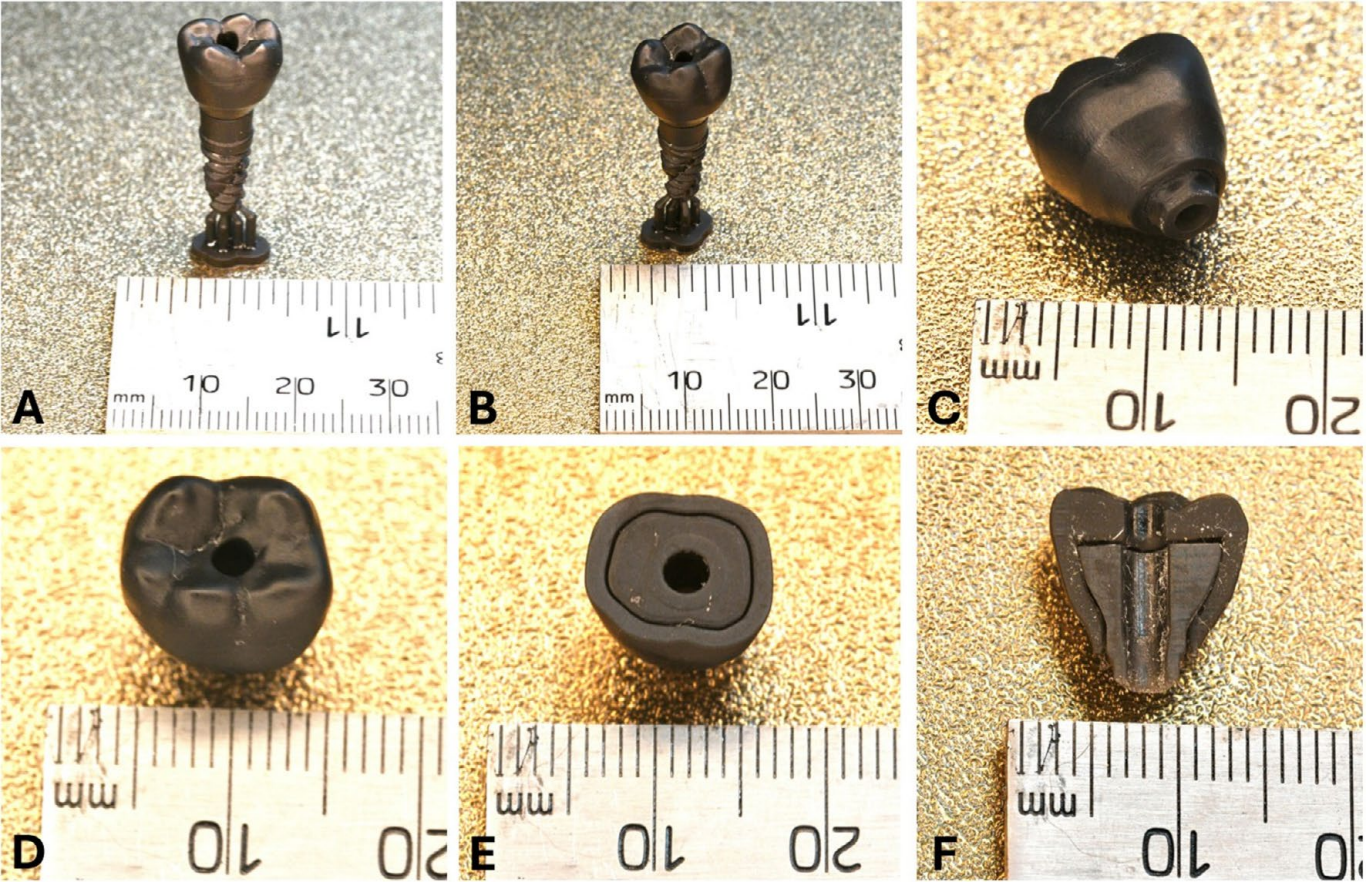
Prototype manufacturing of new design with SLA printer. (A) Crown, abutment, and implant models (lingual view). (B) Crown, abutment, and implant models (buccal view). (C) Implant interface region of restoration. (D) Occlusal surface of restoration with screw channel. (E) Partially completed crown with abutment inserted in cavity during printing. (F) Cross‐section view of crown showing abutment embedded inside.

Detailed pictures of the resulting prototype made on the SLA printer are shown in Figure 17A–F. Visuals of the crown and abutment connected with a model implant are shown in Figure 17A,B. A view of the abutment and crown interface surfaces with the implant fixture is shown in Figure 17C, and the abutment engaging surface protrudes from the bottom of the crown as expected. The occlusal view of the finished screw‐retained crown is shown in Figure 17D. Figure 17E displays the restoration without the top portion of the crown that is normally printed over the abutment. This image is representative of the view after the abutment is inserted during the printing process, but before the printing is resumed. Figure 17F is a cross‐section of the optimized restoration in the buccal‐lingual plane to exemplify how the abutment is embedded inside the crown. While the fit of the abutment inside the crown was not numerically characterized, there is no relative movement between the crown and abutment in the model.

## 4 Discussion

The results from the study support the hypothesis that the new optimized design improved survivability compared to the traditional design while mitigating the debonding failure mode; therefore, the null hypothesis is rejected. This is evidenced by the lower peak Von Mises stress found in the new design compared with the traditional screw‐retained restoration under the vertical and angled loads. Since the traditional restoration has an insert‐style abutment, the load is focused onto a very small area along the mesial side of the upright cylindrical portion that interfaces with the crown due to the thin cross section in the region. Failure testing of screw‐retained Ti‐Base abutments in another study reported this same location as a major failure mode, which corroborates that the traditional screw‐retained Ti‐Base restoration has this survivability drawback [46]. While the new optimized and non‐optimized designs also faced increased Von Mises stress along the mesial face, the larger surface area of the design helps to distribute the load across the interface and maintain lower peak stresses than the traditional design. Additionally, while the maximum principal strain is not much lower on the new optimized design compared to the traditional design, there are hardly any large strains found along the crown and abutment interface. In contrast, there are significant principal strains found in the adhesive along the joint of the crown and abutment in the traditional design.

In the FEA results under the upward load shown in Figure 16A–C, it is clear that the new optimized design has a reduced opportunity for separation of the crown and abutment compared with the traditional screw‐retained restoration. The high shear strain (E13 direction) along the mesial distal surfaces in the occlusal direction on the traditional design will have a very large capacity to shear the adhesive used between the Ti‐Base and the crown, since the adhesive is the only material holding these together. The new design had significantly reduced shear strain along the same surfaces, explained by the embedded style of abutment. Only the Ti‐6Al‐4 V abutment and ceramic resin crown are pushing or sliding against each other in this load case with little shear strain on either. The abutment is firmly embedded and secured inside, alluding to the strength of the connection between the two without the use of adhesive. Where this aspect may stand out clinically is in patients with a small interocclusal space in the posterior region. Small interocclusal space already constrains the height of the crown and abutment, which in turn constrains the effective surface area available to bond the two together in the Ti‐Base screw‐retained restoration. The decrease in height and surface area reduces the force required to debond the two materials [5]. Conversely, the new optimized design can still maintain a strong, mechanically embedded connection between the crown and abutment for this type of patient. Furthermore, the proposed design can help clinicians develop alternative options for single implant‐supported crown restorations where debonding poses a large risk; subsequently, rebonding or repair of crown restorations can be further minimized.

Figure 11A,C highlight that the optimization process improved the new proposed design from the starting restoration before optimization. Even though maximum stiffness was chosen as the objective function and plotted in Figure 11A,C, the method used in this study allowed for a variety of different performance objectives such as stress or mass to drive the optimization if desired. Future studies could instead even target a specific value instead of extrema like in this study, to tune the result to the patient's unique situation. The optimized geometries in Figures 12 and 13 seem to fill outwards to the maximum extent at the top. Tooth crowns naturally have a diverging shape in the occlusal/facial direction, so it makes sense to have an abutment that matches this and capitalizes on being the largest size possible. This naturally increases the strength and survivability of the abutment. Furthermore, the optimization does not seem to completely fill out the available space lower down on the abutment. This could possibly be due to the advent of higher stresses being introduced, in which the stress constraint would force the optimization to avoid expanding metal material to there. Nonetheless, since the maximum stress was below failure stresses of both materials for almost all of the iterations, neither the metal nor the ceramic resin appeared to ever be at risk of permanent deformation in this model under the mastication loads. The stresses appear to focus most at the base of the abutment where the moment exerted on the components by the angled force is largest, similar to other results reported [28]. Extending beyond the scope of the current study, the shape optimization strategy is adaptable to various shape parameters of dental prostheses and can be integrated with digital dentistry workflows reaching beyond implantology.

The results shown in Figure 17 imply that the new optimized design is able to be created for a patient in a clinical setting. A small scar is visible on the external crown surface at the layer where the print was paused, but this can be sanded and polished like any other manufacturing blemish on the surface of a crown. While the form and fit prototyping done in this study did not use the true biocompatible Ti‐6Al‐4 V and ceramic resin materials as designed, some discussion can be made on the feasibility of design and manufacture of this type of restoration for a patient. The crown geometry and implant planning for the patient can be created digitally as usual within a dental software of choice. The proper abutment can also be chosen at this step, but only the portion that interfaces with the implant will be kept. The lower abutment and full crown geometries are uploaded to the nTop software, and the optimization pipeline can be run similar to this study. The optimized abutment result can then be cast, machined, or 3D printed from Ti‐6Al‐4 V using dental lab equipment, and would follow a very similar process to making a custom abutment for an implant restoration. Once this step is completed, the results for the crown are then exported to the 3D printer software, sliced as usual with the screw channel perpendicular to the build plate, and printed with normal parameters for a dental ceramic resin crown material. A Formlabs printer proved to be desirable for this new design and was used in the study. This is because it allows the print to be paused at the opportune layer, the build plate removed, and the abutment inserted as described. Then the build plate can be replaced on the printer, and the print resumed without any issues or red flags in the printer software. There are not any printers on the market that can complete this process automatically, but in theory, a custom‐designed robotic mechanism could accomplish the task. The overall costs of this process are no more expensive than any other screw‐retained design with a custom abutment. Arguably, it may be cheaper since there is no longer the cost of adhesive. Besides the additional time for the optimization to be run (which is not hands‐on working time) and pausing the print to insert the abutment, this new design also does not significantly add to the production time. The time may even be directly comparable, since the Ti‐Base screw‐retained restoration requires surface preparation and curing time for the resin cement connecting the abutment and crown [15]. If time were truly a constraint, in a chairside restoration for example, a stock abutment with a similar diverging shape to the optimized abutment could be used to bypass the 1–2 h for the optimization to converge. In the event that the crown were to be damaged, the remaining material could be sectioned and removed from the metal abutment. Then a new crown could be 3D printed with the reused abutment embedded once again. If the abutment instead breaks, then both the crown and abutment would have to be manufactured again like with the traditional Ti‐Base screw‐retained restoration.

## 5 Conclusions

Based on the findings of this two‐phase shape optimization study, a few key conclusions can be drawn. First, the failure mode of adhesive debonding present in current single implant crown and abutment designs can be eliminated with low consequences to the strength and survivability using alternate crown and abutment shapes and AM. Second, the adaptable two‐phase material shape optimization developed can be used to quickly explore the design space of this crown and abutment shape and offer a high‐performance design. Third, validation finite element analysis studies show that the proposed design still retains low stress and strain after optimization and surpasses the performance of traditional designs.

Improvements to the present study include increasing the mesh fidelity of the optimization, better material property distributions, and more complex loading conditions. Further physical testing of the design, including the separation strength of the abutment and crown, is needed to determine the true performance of the optimized design.

## Ethics Statement

The authors have nothing to report.

## Conflicts of Interest

The authors declare no conflicts of interest.

## Data Availability

The data that support the findings of this study are available from the corresponding author upon reasonable request.
